# Scientific and clinical implications of genetic and cellular heterogeneity in uveal melanoma

**DOI:** 10.1186/s43556-021-00048-x

**Published:** 2021-08-20

**Authors:** Mark J. de Lange, Rogier J. Nell, Pieter A. van der Velden

**Affiliations:** Department of Ophthalmology, Leiden University Medical Center, P.O. Box 9600, 2300 RC Leiden, The Netherlands

**Keywords:** Uveal melanoma, Heterogeneity, Precursor lesion, Driver mutation, Immune infiltrate

## Abstract

Here, we discuss the presence and roles of heterogeneity in the development of uveal melanoma. Both genetic and cellular heterogeneity are considered, as their presence became undeniable due to single cell approaches that have recently been used in uveal melanoma analysis. However, the presence of precursor clones and immune infiltrate in uveal melanoma have been described as being part of the tumour already decades ago. Since uveal melanoma grow in the corpus vitreous, they present a unique tumour model because every cell present in the tumour tissue is actually part of the tumour and possibly plays a role. For an effective treatment of uveal melanoma metastasis, it should be clear whether precursor clones and normal cells play an active role in progression and metastasis. We propagate analysis of bulk tissue that allows analysis of tumour heterogeneity in a clinical setting.

## Introduction

### Background and historical perspective of cancer

The study of cancer dates back as far as centuries B.C. but it was not until the seventeenth century A.D. that research was properly documented and uncontrolled growth of body cells was described [1]. Over the course of centuries, one attempted to unravel the biology of different cancers to get a better understanding and ultimately improve treatment [2]. The first observations were merely morphological, but even before the functional discovery of DNA (1952), the understanding has been that cancer is a genetic disease, which allows for a better understanding of the biology and assessment of its behaviour [3].

**Fig. 1:**
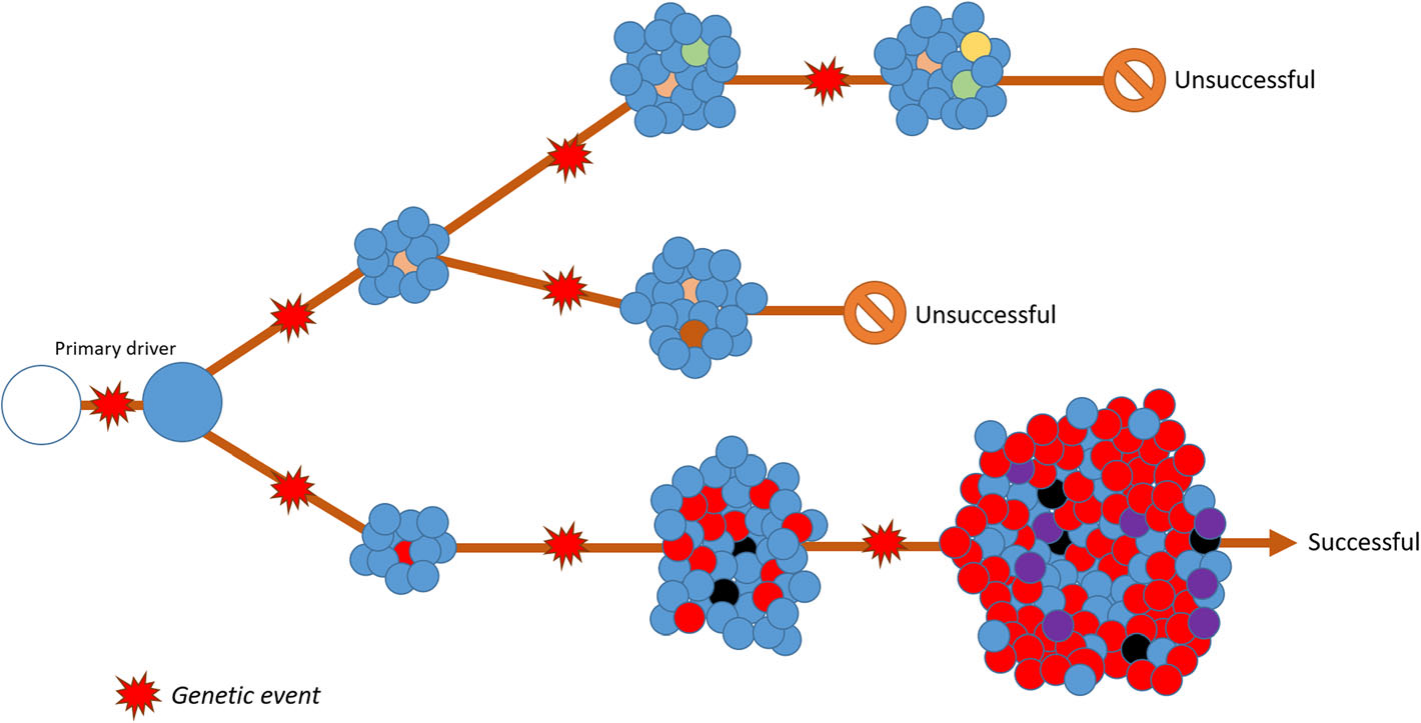
Schematic representation of tumour development. Starting with one primary mutation, which differentiates healthy cells (empty circle) from an early tumour lesion (blue circle), many subsequent mutations are possible. The parental mutation will be present in all daughter cells (blue), while sub-clones are characterised by additional mutations. Non-tumorigenic mutations (beige, green, brown and yellow) do not lead to increased tumour growth, while tumorigenic mutations (red, black and purple) lead to successful tumour development. There are multiple ways a tumour can be unsuccessful, but there are limited ways to be successful

To understand the dynamics of cancer progression, it is important to realise that its development is based on somatic evolution [4]. Tumour growth, differentiation and ultimately metastatic dissemination are driven by genetic alterations. By chance and selection pressure it follows a specific path of mutations and chromosomal aberrations, which eventually lead to a cancer-like phenotype [5, 6]. This process of carcinogenesis can take decades to develop and continues even after the tumour is well formed [7]. One can appreciate that with every accumulating mutation, a unique cell is formed which is different compared to its progenitor though it shares all the pre-existing mutations (Fig. 1).

The resulting diversity, referred to as heterogeneity, is thus present in the very early stages of cancer development and may progress in late progression stages and metastasis [8, 9]. Since the development of cancer is dependent on evolution, every accumulating mutation that occurs in a single cell, and provides an evolutionary benefit over another, will multiply to form a novel clone [10, 11].

Depending on growth advantage of the advanced clone, precursor clones may remain visible. This co-occurrence of precursor clones will allow for the analysis of tumour evolution. In cutaneous melanoma (CM) this has been elegantly demonstrated by dissection and analysis of mixed tumours that contained visible remainders of precursor lesions [12]. However, even if these remains of precursor clones are not histologically visible, they may still be demonstrated by molecular analysis of tissue.

### Uveal melanoma as a model to study heterogeneity

**Fig. 2:**
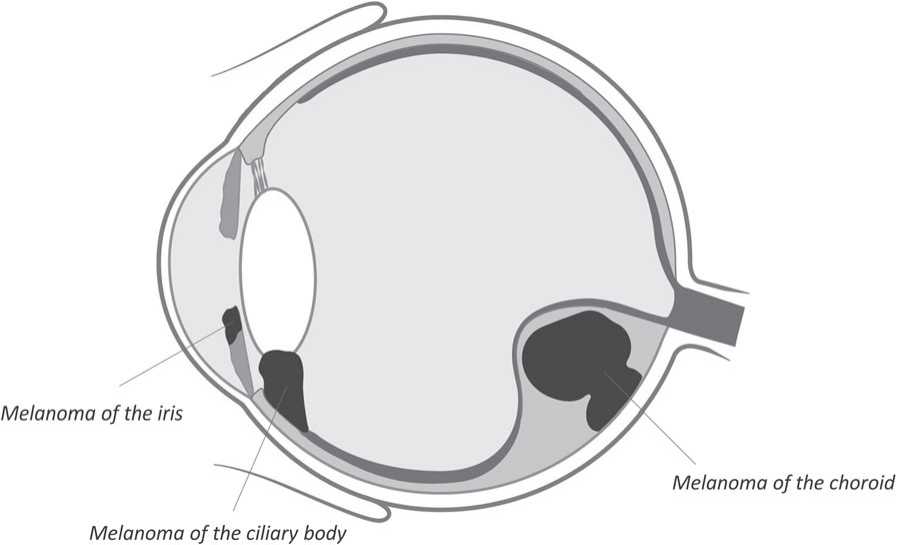
Anatomy of uveal melanoma. (Modified from the figure of Diana Marques for UM Cure 2020, has gotten the permission) [13]

**Fig. 3:**
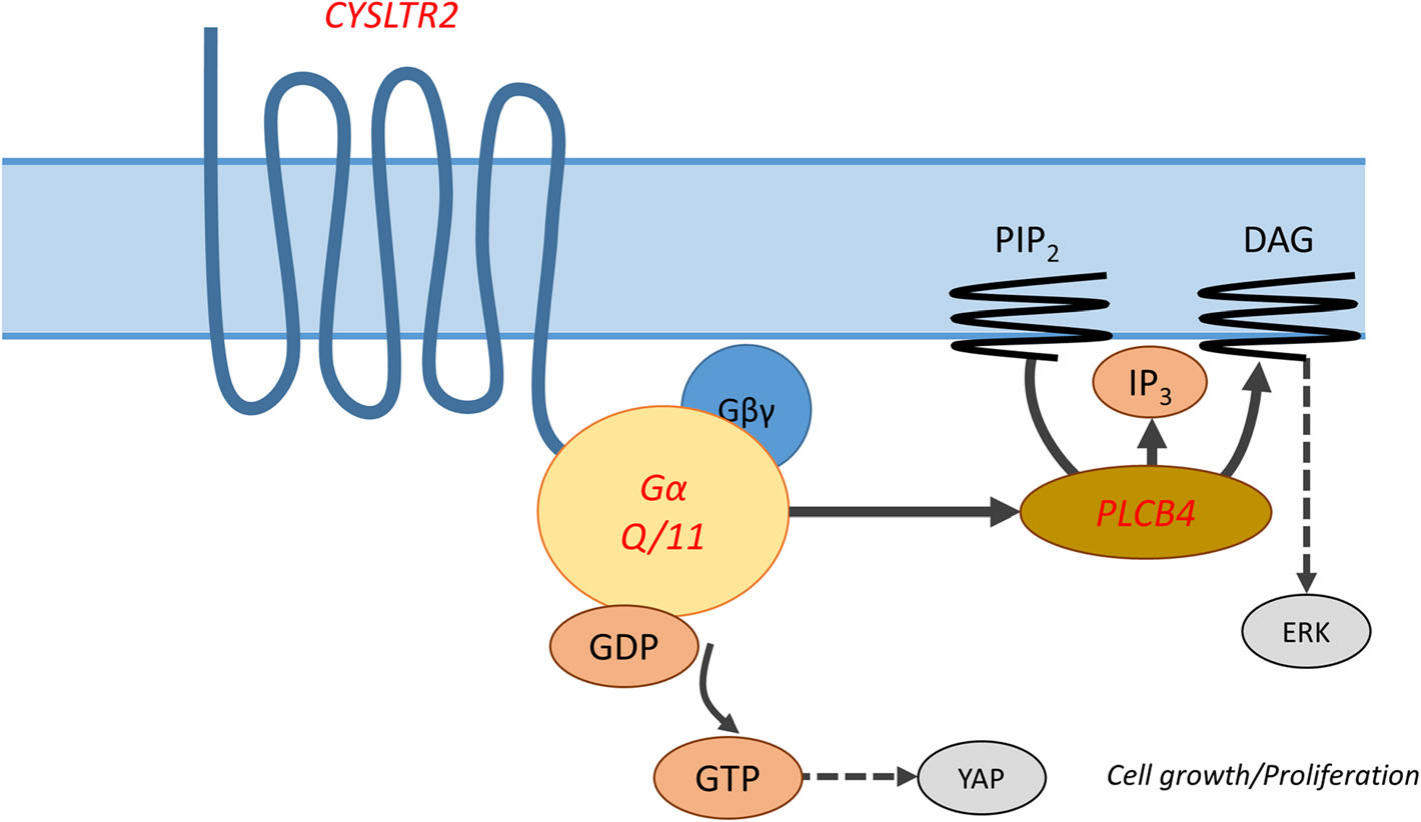
Gαq signalling in uveal melanoma is achieved by mutations in *CYSLTR2*. *GNAQ/11* and *PLCB4*. (Modified from the figure in [20])

Uveal melanoma (UM) is an intraocular neoplasm originating from melanocytes in uvea, which comprises the iris, ciliary body and choroid (Fig. 2). UM is a rare tumour and has an incidence varying from 0.31 to 8 per million individuals, depending on sex, race and country [14]. Similar to CM, there is a distinct possibility that UM are formed out of nevi [15, 16]. The most commonly found mutations in UM and CM, the so-called driver mutations *GNAQ/11 Q209L/P* and *BRAF V600E* respectively, are already present in otherwise healthy nevi and are generally not capable of developing into cancer without additional mutations [16–19]. The important role of Gαq signalling in the development of uveal melanoma has been further supported by the identification of recurrent mutations in PLCB4 (p.D630, encoding phospholipase C β4) and CYSLTR2 (p.L129Q, encodinga Gαq-coupled receptor) [20, 21]. These mutations are typically found in GNAQ and GNA11 wild-type tumours and provide an alternative way to activate the Gαq signalling pathway [20–26] (Fig. 3). One could argue that the presence of these pivotal mutations in melanoma make the tumour genetically homogenous since the same driver mutation may be identified within one tumour (i.e. intratumorally) as well as between tumours found in different patients (i.e. intertumorally). Still, each melanoma originates from a unique somatic genetic event, and this is most dramatically illustrated by patients with multiple choroidal nevi. Though they all present with a Gαq signalling mutation, the studies by Vader et al. and Nell et al. showed that by chance each nevus carries another specific variant [19, 26]. After such driver mutation, additional genetic events are necessary to progress and with each additional event a possible heterogeneity emerges.

Uveal melanoma (UM) provides the ideal model to study heterogeneity since it grows in the corpus vitreous, providing a unique pure cancer-cell environment, whereas in other cancers the interference of tissue that is not related to the tumour is common. UM have a slow growth rate with doubling times ranging from 128 to 292 days depending on cell composition [27]. This makes it more likely to capture preceding clones and thereby allows the study of the heterogeneity of the tumour. Taking the driver mutations as central starting point, heterogeneity in UM can be studied both on a cellular and molecular level. Non-tumour cells can be identified by the absence of this mutation, while tumour cells will always carry it.

### The development of uveal melanoma

#### Secondary mutations in uveal melanoma development

The development of cancer is a multi-step process which has been described in numerous tumour types, and melanoma development should be viewed in a similar fashion [28, 29]. Similar to CM, UM requires so-called second-hit-mutations to progress from a nevus into a cancer-like phenotype [30]. In CM, mutations in the *CDKN2A* gene are candidates for the next step in melanoma development [31–34]. For UM however, this is less evident. Recurrent mutations in *BAP1*, *SF3B1* and *EIF1AX*, have been found in UM but none are as common as the presence of *GNAQ/11* [35–44].

This shows that there are multiple second hits possible, allowing the tumour to divert into different directions and become (intertumorally) heterogeneous with distinct clinical and pathological behaviour. In contrast, intratumorally UM are considered to be homogeneous in a sense that every tumour has its own set of specific mutations [45, 46]. To unravel heterogeneity, phylogenetic tools were used to create a molecular ancestral history of individual UM, which in turn led to the reconstruction of a sequence of events in tumour evolution [45]. This approach is based on the hypothesis that starting from a healthy melanocyte, several sequential ‘hits’ are required to become a successful tumour cell.

**Fig. 4:**
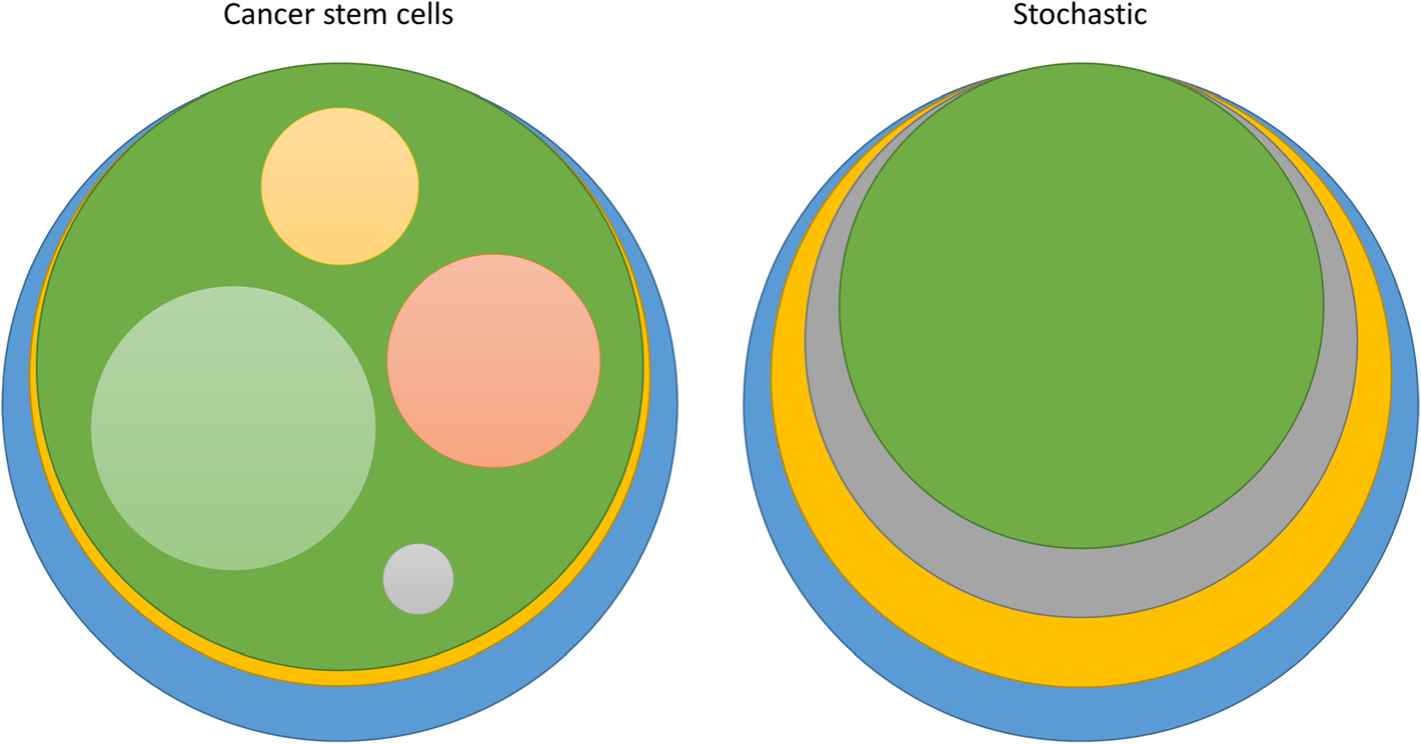
Different models of tumour development and the consequential tumour composition. Each circle represents a pool of cells with identical genetic alterations. In a cancer stem cell model, a vast pool of mutated stem cells produces daughter cells carrying its progenitor’s mutations and mutations unique for that clone. Characteristic for this model is the presence of multiple coexisting clones in the tumour mass. In a stochastic model, cells follow a specific path of mutations leading to increasingly smaller fractions which carry all its progenitor’s mutations. Sub-clones carrying different sets of mutations are unlikely in this model and thereby this model best reflects what we observe in UM

However, the possibility that UM development can follow different paths, even within one tumour, that lead to successful tumour formation makes the presence of heterogeneity within a tumour likely. This intratumour heterogeneity can be viewed in different ways. If we assume the existence of cancer stem cells (CSC), this pool of cells hierarchically governs the fate of the tumour. Typically, this would mean a high turnover of clones that all share a large portion of genetic aberrations with their parental CSCs [9]. This way, clones would come and go and would not operate as separate entities. The consequential heterogeneity of the CSC model is not observed in UM and makes it an unlikely mechanism for this tumour type [47] (Fig. 4). Still, several studies showed the existence of cell populations carrying stem cell like markers in UM and UM cell lines [48–50]. Functional analysis of stem cell marker-positive cells has to tell whether they actually present stem cell-like properties and are able to recapitulate the tumour phenotype [50].

Alternatively, recent research in UM indicated a more linear evolution that still results in different subpopulations that represent different progression steps within one UM [51, 52]. Separate clones would have their own evolved set of genetic mutations and are, depending on the Darwinian model, more or less potent than one another [53].

Given its slow growth rate, progenitor clones are less likely to get completely overgrown by descendant clones. By means of next generation sequencing of spatially separated biopsies, it has already been possible to detect several sub-populations within one tumour [52]. With a dedicated approach like digital PCR, it is even possible to accurately quantify remaining precursor clones in bulk DNA from a single biopsy. The high precision of this technique allows for the comparison of cell fractions from precursor and descendant clones, thereby revealing the evolutionary history of the tumour. For a thorough analysis, it is of vital importance to take different concepts of genetic heterogeneity into consideration since it may affect tumour classification, survival predictions and even treatment options. Theories of intratumour heterogeneity offer new possibilities in unravelling the biology of UM but potentially complicate the treatment thereof.

#### Secondary copy number alterations in uveal melanoma development

Next to recurrent mutations, other common anomalies were found in the form of altered chromosomal copy numbers. In UM, especially chromosome 1, 3, 6, 8 and 16 are affected and are just as common as mutations like *BAP1*, *SF3B1* and *EIF1AX* [54–63]. Chromosome copy number aberrations are related to progression and can therefore be regarded as another step in carcinogenesis [45]. The most common variations which are correlated with increased risk are gain of chromosome 8 or multiple copies of chromosome 8q and loss of one copy of chromosome 3 [64–67]. Early studies that used karyotyping and more advanced in situ chromosome analysis already suggest that chromosomal aberrations within the same tumour do not have to be present in every tumour cell [68–71]. Using advanced quantitative analyses of bulk DNA, this concept of heterogeneity was further investigated [51]. Indeed, some chromosomal changes were only present in a subset of cells within the tumour rather than the tumour as a whole. Very recently, single-cell sequencing confirmed this heterogeneity [72]. Given the previously described path of carcinogenesis, it is far more likely that several clones representing precursors and descendant clones co-exist within one tumour. Hence, UM cannot simply be classified as having a certain aberration, disregarding the proportion of cells this aberration can be found in.

Besides scientific implications, this concept also has possible clinical consequences. Often, correlations between genetic aberrations and survival are made to demonstrate the clinical relevance of molecular biology of UM [65, 73–75]. Gain of chromosome 8q and monosomy 3 are strongly correlated with a bad prognosis while gain of chromosome 6p seems to be almost exclusively correlated to a good prognosis [75].

Absolute quantification of clones also offers opportunities to quantify precursor and descendant clones [51]. This concept thereby allows for defining a sequence of events in UM based on the percentage of cells carrying a specific aberration. Events occurring with higher percentages precede the ones with a lower fractional abundance. Since driver mutations (usually *GNAQ/11*) are present in all tumour cells of most UM, this is considered to be the primary event. As opposed to next generation sequencing, absolute quantification with digital PCR allows to detect small differences in clone fractions and is therefore able to differentiate between early events [26].

#### From primary tumour to metastatic disease

Though intuitively one may assume that the most progressed tumour cells will successfully disseminate, metastases may be formed at various stages of tumour evolution. Theoretically, multiple metastases may present with a different genetic set up that require different treatment approaches.

Recently, three studies explored the genetic characteristics of matched primary and metastatic UM [52, 76, 77]. Although large similarities were found in the alterations affecting different tumours between patients, a notable genetic heterogeneity was discovered within patients. In several cases metastatic lesions did not harbour alterations which were present in the primary tumour. Apparently, the primary tumour was populated by more evolved clones harbouring additional genetic changes whereas an earlier clone already metastasised.

### Cellular heterogeneity in uveal melanoma progression

**Fig. 5:**
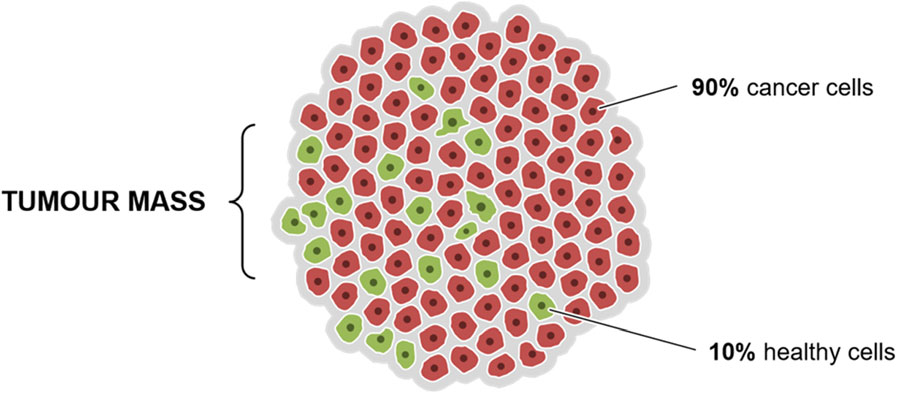
Composition of an average UM. The majority of cells are mutated cancer cells, while a smaller portion consists of non-mutated healthy cells. Since UM grows in the acellular corpus vitreous, all these cells are part of the tumour. (Percentages are hypothetical but representative as described in De Lange et al. [79])

UM grow into the acellular corpus vitreous and are therefore considered to have a high tumour purity (i.e. low stromal cell contamination). However, next to the fraction of cells in a tumour that have genetic aberrations, there is also a part that does not contain genetic defects, often referred to as the tumour microenvironment [78](Fig. 5).

This portion of non-malignant cells can strongly differ between UM [79, 80]. The majority of these cells originate from the immune system [81–83]. The role of the immune system in cancer development is still a topic of debate since the interaction between cancer and the immune system is an evolving process [84, 85]. In some tumour types, the immune system is able to target the mutated cells (anti-tumorigenic), while in other tumour types it apparently supports the tumour’s growth and expansion (pro-tumorigenic) [86–91]. As part of the natural process, natural killer cells, CD8+ T cells, CD4+ T cells, cytotoxic macrophages and neutrophils work together to eradicate aberrant neoplastic growth. This is a concept referred to as cancer immunosurveillance [92–94]. However, when the cancer, due to unknown causes, is successful in avoiding destruction by the immune system, it is hypothesised that tumour-associated inflammatory cells contribute to tumorigenesis by inducing cell proliferation and immunosuppression [94–96]. For UM however, the role of the immune system remains poorly understood. It is unclear whether the immune system can recognise tumour cells as foreign, or that it is actively recruited by tumour cells to aid in tumour progression. Epidemiologically, numerous studies have shown strong correlations between the infiltration of innate and adaptive immune cells on one hand and the presence of genetic abnormalities and increased metastatic risk on the other hand [97–99]. However, the mechanism of how the immune system is involved remains unclear and might simply reflect co-occurring events [51]. Ironically, an inflammatory response in UM may even be perceived as uveitis, a phenomenon referred to as masquerade syndrome [100, 101].

In the healthy state, the eye is considered to be an immune privileged organ, meaning that the blood retinal barrier (BRB), similar to the blood brain barrier (BBB), acts as a physical barrier for both pathogens and immune cells [102–104]. This prevents unnecessary immune reactions in an organ with nonregenerative delicate tissue, protecting it from harm and loss of vision [105]. However, this specifically applies to the vitreous fluid that is in contact with the retina. The choroid on the other hand is separated from the retina by the Bruch membrane and therefore can be considered to represent another compartment. Hence the extent to which immune privilege applies to the choroid is questionable [106].

**Fig. 6:**
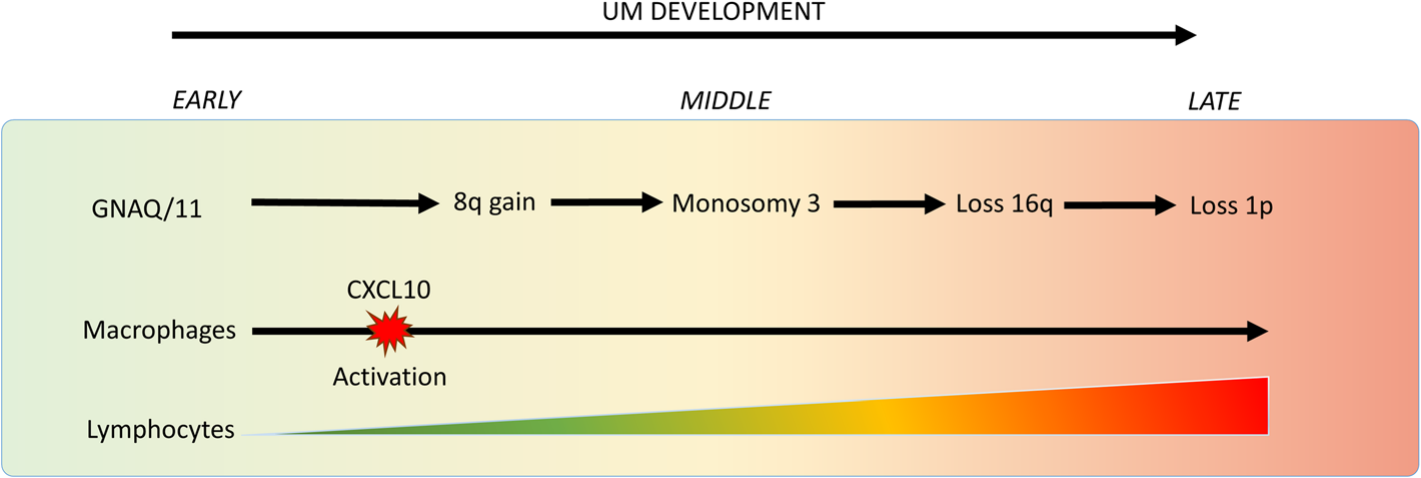
UM development where multiple processes within a UM occur simultaneously. UM progression coincides with macrophage activation while the consequential chemokine expression (CXCL10) of activated macrophages is correlated with an influx of lymphocytes

As a result, lymphocytes have access to the choroid, and they do appear in UM. This still suggests that an active influx of T-cells is possibly triggered by tumour growth. The resulting cellular heterogeneity has implications for comprehending UM dynamics that lead to tumour progression and ultimately metastasis. Unfortunately, it is not known what the cause and the resulting effect is, which would be helpful in clarifying what role the immune system plays in UM development. Can we measure the immune infiltrate and thereby predict tumour behaviour for diagnostic, prognostic and eventually treatment purposes? Historically, the presence of lymphocytes and macrophages was shown histologically [107, 108]. More recent approaches involve cytometry in combination with mass spectrometry and single cell genomics to analyse complex tissues. In CM these approaches revealed that lymphoid structures and lymphocytes play an important role in the response to immunotherapy [109, 110]. In UM, in contrast, the lymphocytes were correlated to an immune-suppressive environment and an unfavourable outcome [72, 111]. Recent research shows that, by using digital PCR and tumour DNA, it is now possible to generate an absolute T cells count in UM [79, 112]. Absolute quantification of immune cells in bulk tissue of UM facilitated the deconvolution of the microenvironment and revealed a prime role for the macrophages in the establishment of the immune infiltrate [79]. Moreover, using genetic evolution of UM as a molecular clock to analyse the development of the immune infiltrate we could show that macrophage invasion actually precedes T cell influx [113]. The expression of chemokine CXCL10 by macrophages was highly correlated to T cell numbers and suggested that this chemokine is instrumental in UM inflammation. CXCL10 expression was part of an extended expression profile that characterised macrophages that were treated with classic activators (Figs. 6, 79]. These results are thereby in contrast to reports that suggest that macrophages of the M2 phenotype are most prominent in inflamed UM [97].

Detailed knowledge on the immune microenvironment may reveal opportunities in uveal melanoma treatment. The therapies that have been successful in cutaneous melanoma rarely are effective in uveal melanoma [13, 76]. The massive T-cell infiltrates that can be observed in uveal melanoma could represent an opportunity for immune therapy. It is, however, the question whether the metastatic uveal melanoma evokes a similar immune invasion that could be redirected for therapeutic purposes.

In UM metastasis, the immune invasion is not as prominent as in the primary UM and often limited to the margins of the tumour [114]. This questions whether there is a true interaction between UM and immune cells or if it reflects local immune environment that is governed by tissue-specific mechanisms (Fig. 4).

## Discussion

It is impossible to speak about a standard tumour with standard characteristics. Just as our bodies differ from one another, determined by our genetic code, every tumour has its own identity. Although uveal melanomas could be considered genetically homogeneous based on their shared Gαq signalling mutations, occurring in almost all uveal melanomas, subsequent genetic alterations driving tumour progression cause genetic differences between tumour sub-clones.

A tumour mass, especially a primary uveal melanoma, should not be seen as a homogenous clump of cells. Although some alterations may be shared between all cancer cells, others are only present in distinct clones and thus a subset of the cells. However, the clinical significance of intratumour heterogeneity in UM has not yet been fully revealed. But it is clear that different clones can metastasise from a heterogeneous tumour, and that this may require adjustments in the treatment of metastatic disease [52]. Moreover, genetic aberrations that are correlated with bad prognosis in the primary UM are possibly unrelated to disease progression in the metastasis [115]. Apparently, the requirements in the metastasis are different from the requirements in the primary tumour. The exception to the rule seems to be 8q gain that appears to be deduced to a positive selection pressure and commonly enriched in the metastasis [52].

When different clones coexist within one tumour, the spatial distribution of the clones may influence the detection of their presence. When a certain clone contains prognostic or therapeutically relevant genetic information, being able (or unable) to identify its presence has immediate clinical consequences. On the other hand, the presence of non-cancer cells should always be taken into account. Apart from its biological relevance, its influence on the detection of genetic alterations is inevitable as it ‘contaminates’ the tumour with a fully healthy genome. A major challenge comes in the exact differentiation of copy numbers. Whereas mutations can be identified as newly acquired changes, never present in any other cell, copy number alterations may present as very limited imbalances [66].

By the analysis of different progression stages of UM, it was shown that metastases may actually be derived from precursor clones and not necessarily from the last progression step of the primary UM. This shows again that UM not only grow slowly but also progress slowly. Years after treatment of the primary UM, liver metastases may arise that were not present at the time of treatment. To consider that spreading by a precursor clone would then already have occurred, puts progression and metastatic spreading of UM in a whole new perspective. Detailed knowledge on the lesions present in the primary UM and the UM metastasis and the order in which they occurred is of importance for the molecular evolution and the possibilities for treatment. Eventually this should indicate possibilities to prevent metastases. A full understanding of all the steps in metastasis will eventually allow the development of modalities to inhibit the correlated pathways and prevent metastasis. Monosomy 3 for example defines tumours with a poor prognosis. Inactivation of the tumour suppressor genes *BAP1* and *RASSF1A*, localised on chromosome 3, accompanied by activation of the *MYC* oncogene on chromosome 8q may follow this loss of chromosome 3 and could even be the cause of poor survival [59]. Recent research supports that UM is a dynamic tumour that most likely cannot be cured by single target therapies [116, 117]. Moreover, epigenetic mechanisms are not taken into account in this review but do add another layer of complexity that also has to be considered [118]. Unravelling the molecular background of UM in order to develop appropriate personalised treatment is therefore of vital importance. As mentioned, heterogeneity is not only present between tumours but also within one tumour. The genetic landscape and cellular microenvironment, differing from one tumour to another, both influence tumour growth and should therefore be taken into account when determining prognosis and treatment.

## Data Availability

NA. This is a review and no original data is shown.

## Abbreviations

BBB: Blood brain barrier
BRB: Blood retinal barrier
CM: Cutaneous melanoma
CSC: Cancer stem cell
PCR: Polymerase chain reaction
UM: Uveal melanoma
